# Exogenous hydrogen sulfide exerts proliferation, anti-apoptosis, migration effects and accelerates cell cycle progression in multiple myeloma cells via activating the Akt pathway

**DOI:** 10.3892/or.2021.7923

**Published:** 2021-01-05

**Authors:** Dong Zheng, Ziang Chen, Jingfu Chen, Xiaomin Zhuang, Jianqiang Feng, Juan Li

Oncol Rep 36: 1909-1916, 2016; DOI: 10.3892/or.2016.5014

Following the publication of this article, an interested reader drew to the authors’ attention that, in Fig. 4 on p. 1913, the t-Akt panel in Fig. 4A looked unexpectedly similar to the β-actin panel in Fig. 4C. The authors were able to refer back to their original data, and realized that the Figure had been compiled incorrectly; essentially, the data for the t-Akt panel had been duplicated, and the data for the β-actin panel in Fig. 4C had not been included in the Figure as intended.

The revised version of Fig. 4, showing the correct data for the β-actin panel in Fig. 4C, is shown opposite. This error did not have a significant impact on the results or the conclusions reported in this study. The authors are grateful to the Editor of *Oncology Reports* for allowing them the opportunity to publish this Corrigendum, and all of the authors agree to the publication of this Corrigendum. The authors sincerely apologize for this mistake, and regret any inconvenience this mistake has caused.

## Figures and Tables

**Figure 4.**
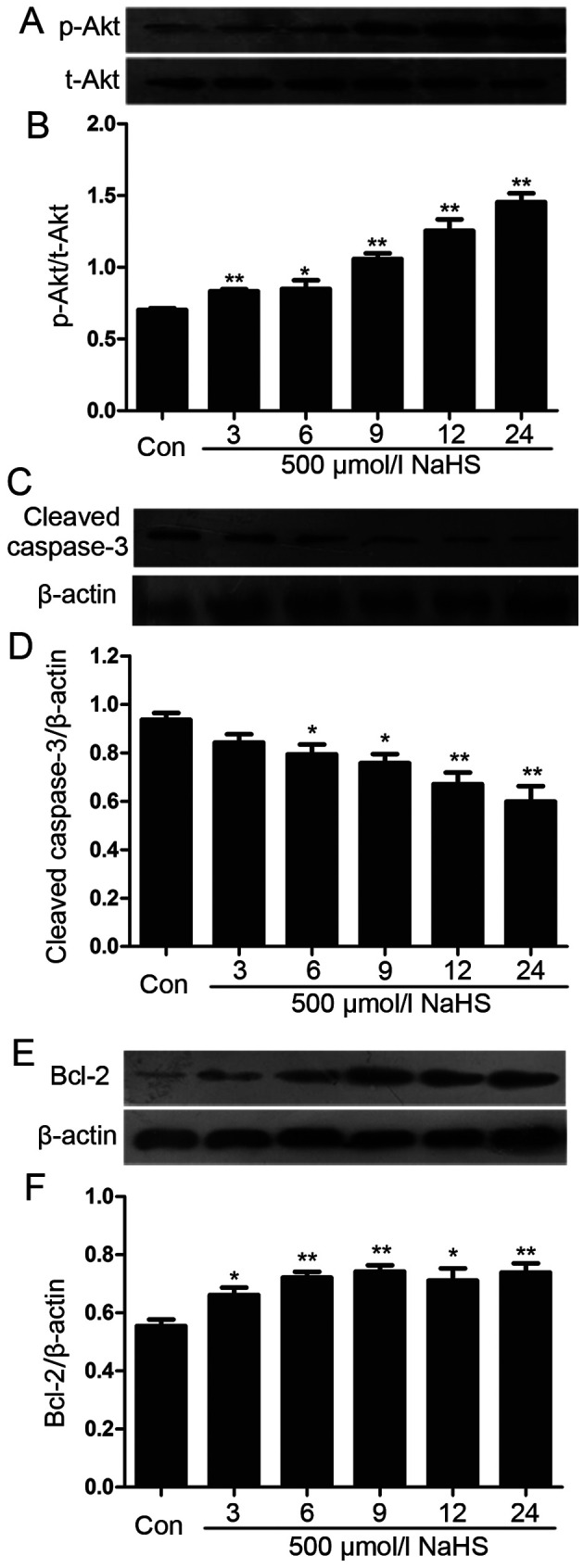
NaHS amplifies the activation of Akt via phosphorylation in myeloma cells. Myeloma cells were exposed to 500 µmol/l NaHS for the indicated times (3, 6, 9, 12 and 24 h). NaHS reduced the expression of caspase-3 and upregulated the expression of p-Akt and Bcl-2 in myeloma cells. The expression of caspase-3 and Bcl-2 was measured using western blot analysis (B, D and F). The data shown in (A, C and E) were quantified using densitometric analysis in ImageJ 1.47. The data are shown as the mean ± SEM (N=3). *P<0.05, **P<0.01 compared to the control group. Con, the control group; NaHS, a donor of H2S.

